# Association of Mediterranean Diet Adherence with Sociodemographic, Anthropometric, and Lifestyle Factors during the COVID-19 Pandemic: A Cross-Sectional Study in Greece

**DOI:** 10.3390/nu15194123

**Published:** 2023-09-24

**Authors:** Eleni Pavlidou, Sousana K. Papadopoulou, Maria Mentzelou, Antonios Dakanalis, Theofanis Vorvolakos, Georgios Antasouras, Maria Spanoudaki, Aimilia-Lynn Pandi, Aspasia Serdari, Maria Chrysafi, Sofia Dimoliani, Constantinos Giaginis

**Affiliations:** 1 Department of Food Science and Nutrition, School of the Environment, University of the Aegean, 81400 Lemnos, Greece; elen.p.pavl@gmail.com (E.P.); maria.mentzelou@hotmail.com (M.M.); g.antasouras@gmail.com (G.A.); fnsm22020@fns.aegean.gr (A.-L.P.); m.chrisafi3@gmail.com (M.C.); sdem@aegean.gr (S.D.); 2 Department of Nutritional Sciences and Dietetics, School of Health Sciences, International Hellenic University, 57400 Thessaloniki, Greece; souzpapa@gmail.com (S.K.P.); maryspan1@gmail.com (M.S.); 3 Department of Mental Health, Fondazione IRCCS San Gerardo dei Tintori, 20900 Monza, Italy; antonios.dakanalis@unimib.it; 4 School of Medicine and Surgery, University of Milano-Bicocca, 20900 Monza, Italy; 5 Department of Geriatric Psychiatry, School of Health Sciences, University General Hospital of Alexandroupolis, Democritus University of Thrace, 68100 Alexandroupolis, Greece; tvorvola@med.duth.gr; 6 Department of Psychiatry and Child Psychiatry, School of Medicine, Democritus University of Thrace, 68100 Alexandroupolis, Greece; aserdari@yahoo.com

**Keywords:** COVID-19 pandemic, quality of life, sleep quality, anxiety, depression, sociodemographic parameters, anthropometric parameters, lockdown

## Abstract

Background: The COVID-19 pandemic has negatively affected several aspects of people’s lifestyle worldwide. Healthy dietary patterns and their bioactive components may improve or even co-treat the negative impacts of the COVID-19 pandemic in several aspects of people’s lifestyle and mental health in daily life. The aim of this survey is to evaluate the potential effect of Mediterranean diet (MD) adherence against COVID-19-induced complications. Methods: This is a cross-sectional survey performed on 3721 adults aged between 18 and 65 years old, which aims to evaluate the potential association of MD adherence with multiple sociodemographic, anthropometric, and lifestyle factors during the COVID-19 pandemic period. Results: This study has supported evidence that elevated MD compliance was independently related to female gender, better economic status, no smoking, increased risk of abdominal obesity, higher physical activity levels, greater prevalence of adequate sleep quality, better quality of life, and reduced probability of anxiety and depression during the COVID-19 pandemic by adjusting for multiple confounders. Conclusions: MD compliance may improve or even co-treat the negative impacts of the COVID-19 pandemic in several aspect of people’s lifestyle in daily life. Further research is strongly recommended exploring the possible beneficial effects of the MD against COVID-19 lifestyle complications in daily life.

## 1. Introduction

The COVID-19 pandemic began at the end of 2019, affecting several aspects of people’s daily life. To prevent the disease from spreading, countries were obliged to enact stringent health regulations and social segregation policies. Particularly, in nations that have undergone protracted containment measures, the social distancing command, teleworking [1], the closing of schools and companies, and general isolation appear to exert a substantial deleterious impact on people’s psychology and daily routines [2]. Thus, the COVID-19 pandemic has become a challenge for governments and healthcare professionals. The significant medical complications, the increased prevalence of morbidity, and the quick international diffusion of COVID-19 have resulted in critical public health concerns, activating several public governmental actions worldwide.

The COVID-19 pandemic has important detrimental consequences on several aspects of mental health, and, thus, the WHO has emergently addressed the mental health of the general population as an issue of great priority [3,4]. Several longitudinal studies have showed that in the first phase of pandemic, mental health (i.e., anxiety, depression, and general distress) worsened compared with the time prior to the pandemic [5]. Recent studies have indicated that during the epidemic, many persons experienced negative emotional impacts because of the worry of infection and of the death of their relatives [6]. In this aspect, the COVID-19 pandemic was a distressing event which included various kinds of stress factors such as worry of infection, fear for family members’ health, social disconnection and isolation, the interruption of routine actions in daily life, and modification in financial status [7,8]. In a substantial population-based cross-sectional survey, social isolation during the COVID-19 pandemic was found to exert a global negative effect on residents’ behaviors [9]. For example, a considerable elevation in the prevalence of sedentariness, alcohol consumption, and smoking was noted. Accordingly, eating habits were considerably worse during the pandemic [9].

Noticeably, the immune system’s function and the probability of interaction with food have attracted specific attention during the effort to limit infection in order to provide a better functional response and more effective protection against COVID-19 [10,11]. Adequate food intake appears to provide some amount of protection against the virus and can assist in managing the infection in the event of illness [12]. It has been well-recognized that when an organism is exposed to an infectious agent, the immune system works much more intensively [13]. This increased action is supplemented by an accelerated metabolic rate, which requires additional energy, higher biosynthetic substrates, as well as regulatory substances derived by foods [14]. A balanced, healthy diet can therefore be instrumental in enhancing the immune system, which is crucial for fighting various infections. In contrast, an unhealthy diet has adverse effects in terms of inflammatory conditions and oxidative stress, and therefore can considerably affect the final outcome by weakening the immune system [14]. Since shortages of specific nutrients from the body (such as vitamins C and D and omega-3 fatty acids) might increase the chance of infection, there is gradually continued interest in immune-boosting supplements that could help to prevent COVID-19 or cure its symptoms [15,16,17]. Many substances have not been fully examined for this disease, although it is known that they may help to prevent or minimize the symptoms of the common cold, influenza, and other respiratory diseases. Therefore, some scientists have believed that they may also be effective against COVID-19 [15,16,17]. Regular vitamin C intake has been demonstrated to lessen the duration and intensity of the common cold, as well as the risk of colds in individuals subjected to significant physical stress. It is also beneficial for those with lung diseases, presenting reduced levels of vitamin C, as well as those with viral illnesses, such as shingles. Vitamin C’s antioxidant properties may also lower oxidative stress during infections [18].

Several research studies have also indicated that vitamin D deficiency and insufficiency are also frequent in the global population [18,19]. Notably, researchers have reported a link between COVID-19 severity and serum vitamin D concentrations in the last two years [18,19,20,21,22,23]. In the setting of COVID-19, 1,25(OH)2D3 seems to downregulate the early viral phase (SARS-CoV-2 infection) by enhancing innate antiviral effector mechanisms, as well as the later cytokine-mediated hyperinflammatory phase [21]. By considering the common origin of vitamin D, cortisol and sex hormones from cholesterol, certain reports of an interaction between the secosteroid 1,25(OH)2D3 and other steroidal hormones are not unexpected, in particular regarding common metabolic and cellular effects on immune system response and autoimmunity [23]. Importantly, 25OH-vitamin D serum deficiency has been associated with more severe lung involvement, longer disease duration, and risk of death in elderly COVID-19 patients. Moreover, the detection of low vitamin D levels in younger COVID-19 patients with fewer comorbidities has further suggested vitamin D deficiency as a crucial risk factor at any age [22]. In addition, key dietary components such as vitamins C, D, and E; zinc; selenium; and omega-3 supplements have been well-established to exert immunomodulatory effects, with special benefits in infectious disease, and COVID-19 patients may show improvements in all clinical complaints except for bodily pain and tiredness, appetite, and olfactory symptoms [23].

During the acute infection and recovery phase of COVID-19, oral probiotics may also lower the antimicrobial resistance reservoir in the gut microbiota, whereas antibiotic therapy has increased the amount of antimicrobial resistance [24,25,26]. These findings have provided new evidence on the dynamics of the antimicrobial tolerance reservoir in individuals with COVID-19 and raise the idea that microbiota-directed therapy could help individuals with COVID-19 who were characterized by accumulated antimicrobial tolerance, reducing their burden [24,25,26].

As a result of the psychological repercussions and restrictions imposed by the lockdown, the pandemic itself led to worsening lifestyle patterns worldwide, including an elevation in the intake of unhealthy foods and a decline in both compliance to the Mediterranean diet (MD) and physical activity [27,28]. The MD mainly includes plant foodstuffs like fruits, vegetables, cereals, legumes, nuts, seeds, and olives [28,29]. Extra virgin olive oil is the main source of added fat and is consumed along with a high to intermediate consumption of fish and seafood (about two servings per week); intermediate weekly intake of eggs, poultry, and dairy products (two servings per week); a reduced intake of red meat (about a maximum of two servings per week); processed meat (about one serving per week); and sweets [27,28,29]. Increased consumption of nutrients which act against inflammation and oxidative stress and also present immunomodulatory properties is produced by this combination of eating behaviors. These nutrients include dietary fiber, unsaturated fats, polyunsaturated fatty acids (PUFAs), vitamins, minerals, and several bioactive phytochemicals, such as polyphenols and flavonoids [27,28,29]. An inverse relationship between MD compliance and the likelihood of cancer, cardiovascular disease, neurodegenerative illnesses, and metabolic disorders has been suggested by several previous meta-analyses [30,31,32]. It was also shown that the MD may reduce the probability of sepsis and lung infections, as well as inflammation, as indicated by a reduction in c-reactive protein (CRP) and proinflammatory cytokines [30,31,32].

The MD has been presented as a feasible method for addressing COVID-19 infection and severity-related issues such as obesity, diabetes, and cardiovascular disease [30,31,32,33]. The MD also exerts a beneficial effect against inflammatory conditions, protecting the immune system, while it may act as a protective agent against serious acute respiratory syndrome coronavirus [34]. However, until now, there is merely a small number of studies which have explored the impact of healthy dietary patterns or individual foodstuff components against COVID-19 infection, and there are even fewer studies exploring the impact of MD adherence against COVID-19 infection [34,35,36,37]. Hence, the present cross-sectional study constitutes one of the few currently available studies that intends to explore the potential effect of MD compliance in various sociodemographic, anthropometric, and lifestyle characteristics like physical activity, sleep quality, quality of life, anxiety, and depression during the COVID-19 lockdown period.

## 2. Materials and Methods

### 2.1. Study Population

In the current survey, 5123 individuals were primarily assigned from 10 geographically diverse Greek areas: rural, urban, and islands (Athens, Thessaloniki, Larissa, Alexandroupolis, Ioannina, Patra, Kalamata, Crete, and South and North Aegean). The inclusion criteria for the primary assignment were individuals, both adult men and women at an equal number, with an age from 18 to 65 years old. Recruitment to the study was performed during the COVID-19 lockdown in Greece in 2020–2021.

All participants’ information was confidential. All participants were notified concerning the purpose of the study and signed a consent form in which they gave their approval to publish their individual data anonymously. Sample size calculation was established utilizing PS: Power and Sample Size calculator program. The randomization was conducted utilizing a sequence of random binary numbers (i.e., 001110110, in which 0 signified assignment and 1 no assignment to the survey).

**Figure 1:**
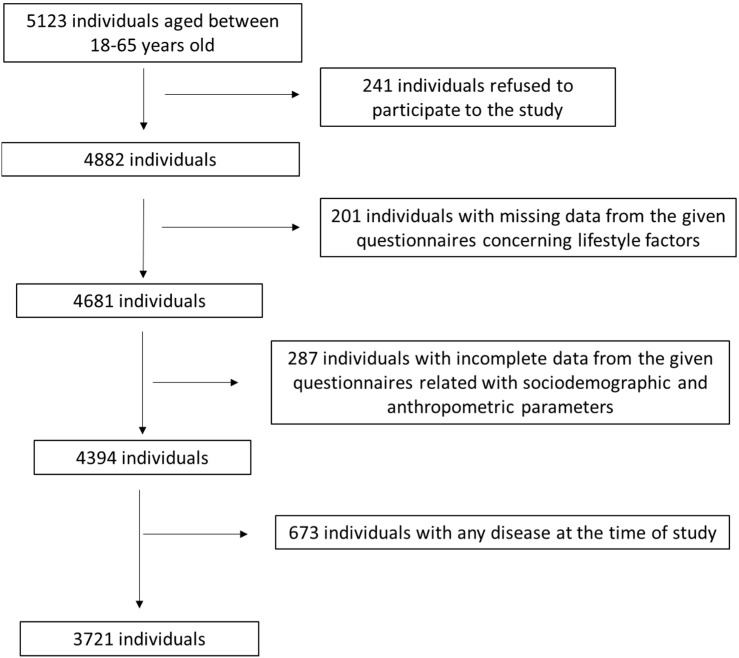
Flow chart diagram of study assignment.

Among 5123 primarily enrolled individuals, 241 (4.7%) of them finally denied taking part in the survey. Among the remaining 4882 individuals, 201 (4.1%) individuals were excluded due to missing data from the given certified questionnaire concerning physical activity, sleep quality, quality of life, anxiety, and depression. Among the remaining 4681 individuals, 287 (6.1%) individuals were excluded due to incomplete data from their given questionnaire related to sociodemographic and anthropometric parameters. Among the remaining 4394 individuals, 673 (15.3%) individuals were excluded from the final analysis due to the presence of any disease at the time of study, such as cardiovascular diseases, cancer, metabolic disorders, and eating disorders. Finally, 3271 adults participated in the final analysis, leading to a final response rate of 72.6%. A flow chart diagram of study assignment is depicted in Figure 1. The study was approved by the Ethics Committee of the University of the Aegean (ethics approval code: no 21/19 December 2019, approval date: 19 December 2019) and it was in accordance with the World Health Organization (52nd WMA General Assembly, Edinburgh, Scotland, 2000).

### 2.2. Study Design

Relevant semi-quantitative questionnaires were used for assembling the sociodemographic data, including age, gender, educational level, family economic status, nationality, living status, employment status, smoking habits, and type of residency, of the enrolled individuals via face-to face interviews between the enrolled participants and the trained personnel to minimize recall bias [38,39]. Education status was estimated based on the summation of the educational years and financial level was categorized based on the yearly family income as: 0, <5000 EUR; 1, 5000–10,000 EUR; 2, 10,000–15,000 EUR; 3, 15,000–20,000 EUR; 4, 20,000–25,000 EUR; 5, >25,000 EUR. Financial level was additionally categorized as low for family yearly income ≤ 10,000 EUR, medium for yearly income ˃10,000 EUR and ≤20,000 EUR, and high for yearly income ˃ 20,000 EUR [38,39]. The above classification was based on the mean annual income of Greek citizens, which is about 19,893 EUR [38,39].

Body weight and height were also determined at the time of study to calculate Body Mass Index (BMI). Participants’ weight was measured using a Seca scale [Seca, Hanover, MD, USA], without shoes, to the near 100 g, while height was measured using a portable stadiometer (GIMA Stadiometer 27335, Athens, Greece) with no shoes on, to the nearby 0.1cm. The WHO recommendations were applied to classify the assigned individuals as normal weight, overweight, or obese [40,41]. A BMI between 18.5 and 24.9 Kg/m^2^ shows a normal weight. A BMI between 25.0 and 29.9 Kg/m^2^ shows overweight, while a BMI 30.0 Kg/m^2^ and above indicates obesity [40,41]. The waist circumference was determined at the midpoint between the lower margin of the last palpable ribs and the top of the iliac crest, while the hip circumference was determined near to the widest portion of the buttocks, with the tape parallel to the floor [42,43]. The Waist–Hip Ratio (WHR) was estimated by dividing waist measurement by hip measurement. WHR has been considered as superior to BMI [44]. It has been recognized as a greater indicator of abdominal obesity [45], which is considered as a better anthropometric measure for more efficiently estimating the probability of various cardiometabolic disorders like diabetes mellitus II [46]. A WHR value of 0.80 or lower for women and 0.90 or lower for men shows low health risk. A WHR value between 0.81 and 0.85 for women and between 0.96 and 1.0 for men shows moderate health risk, while a WHR 0.86 or higher for women and 1.0 or higher for men indicates high health risk [45,46].

We also assessed physical activity levels utilizing the International Physical Activity Questionnaire (IPAQ), in which individuals reported how much exercise they did in a normal week. This self-reported questionnaire is utilized worldwide, assessing the total physical activity over the previous 7 days, to be classified as low, moderate, or high [47]. IPAQ tool has been comprehensively examined and showed high reliability and adequate validity, at least as effective as other self-reported PAQs [47].

We further assessed sleep quality by utilizing the Pittsburgh Sleep Quality Index (PSQI), which contains 19 questions which are scored on a four-point scale (0–3) and classified into 7 components (sleep quality, sleep latency, sleep period, habitual sleep effectiveness, sleep disruption, taking sleeping medicines, and daytime disfunction) [48]. The items’ scores in each component were added and transformed into component scores ranging between 0 (better) and 3 (worse) according to the related guidelines [48]. Total PSQI scoring was determined as the sum of the 7 component scores ranging from 0 to 21, where greater scoring shows inferior sleep quality. An overall PSQI scoring < 5 reveals adequate sleep quality [48].

The World Health Organization’s Quality of Life Questionnaire (WHOQOL-Bref) was applied for assessing quality of life of participants. This is a usual tool for assessing the quality of life in both healthy individuals and patients, which is appropriate to be applied across genders, educational levels, and ages, and is also considered cross-culturally applicable [49]. It has a great validity by giving evidence for adequate convergent and differentiated validity and internal reliability of the physical, psychological, and environmental fields [49]. Each individual item of the WHOQOL-Bref is scored from 1 to 5 on a response scale, which is ordered as a five-point ordinal scale. The scores are then transformed linearly to a 0–100 scale [49].

The 6-item short-form State–Trait Anxiety Inventory (STAI-6) was used to assess the anxiety of participants [50]. This is a consistent and valid instrument with acceptable reliability and validity, as well as precision to variations in state anxiety. It is also likely to maximize responses rates and reduce the number of answering mistakes and unanswered items, therefore enhancing the validity and generalizability of any results [50].

The Beck Depression Inventory (BDI-II) was applied for assessing the depression of the participants. This questionnaire contains 21 groups of statements and is one of the most broadly utilized psychometric tests for determining the intensity of depressive symptoms [51]. BDI-II contains items related to depressive symptomatology like hopelessness and irritability, cognitions like guilt or feelings of being punished, as well as physical symptoms like fatigue, weight decline, and lack of interest in sex [51]. The BDI-II has been considered as a highly appropriate psychometric tool, indicating adequate consistency and capability to distinguish between depressed and non-depressed individuals, and shows increased concurrent, content, and structural validity. According to the available psychometric evidence, the BDI-II is considered as a cost-effective questionnaire for determining the intensity of depressive symptoms, with wide applicability for research and clinical practice worldwide [51].

Concerning MD assessment, the validated MedDietScore was used [52,53]. This questionnaire records the food frequency consumption of eleven selected foodstuff groups based on MedDietScore index. Every question contains 6 probable responses, ranging from 0 to 5, which depend on the level of compliance with each foodstuff group. The summation of the eleven responses results in a score between 0 and 55; a greater score represents elevated MD compliance [52,53]. Concerning cereals, potatoes, fruits, vegetables, dairies, and olive oil, the rates of six possible responses referred to daily consumption. Regarding legumes, fish, red meat, and poultry, the rates of 6 probable responses referred to weekly consumption [52,53]. The 11th question evaluated wine drinking at a daily frequency, with intermediate drinking (≤1 and ≤2 drinks/day for women and men, respectively; one drink = 100 mL = 12 g ethanol) being recognized as the greatest score [52,53]. The enrolled individuals were classified into quartiles according to their MedDietScore. A MedDietScore below 23 shows very low MD adherence, and a MedDietScore between 23 and 26 indicates low MD adherence. A MedDietScore between 27 and 30 shows moderate MD adherence, while a MedDietScore 31 and above indicates high MD adherence.

The questionnaires were accomplished by qualified personnel (e.g., medical and nursing personnel), nutritionists, and dietitians during face-to-face interviews with community-dwelling assigned participants. The qualified personnel explained all the questions in detail to all enrolled adults to increase the accuracy of answers.

### 2.3. Statistical Analysis

Student’s *t*-test and one-way ANOVA were used concerning continuous variables, which followed the normal distribution. Kolmogorov–Smirnov test was applied to assess normality distribution. Categorical variables were evaluated with Chi-square. The quantitative variables showing normal distribution are expressed as mean value ± Standard Deviation (SD). The qualitative variables are expressed as absolute or relative incidences. Multivariate binary logistic regression analysis was applied for examining whether MD compliance is independently related to sociodemographic, anthropometric, and lifestyle characteristics with adjustment for various possible confounders. As confounders, we entered all the available co-variates, as all of them could have a confounding impact. Multiple regression results are expressed as Odds Ratios (OR) and 95% confidence intervals (CI). The Statistica 10.0 software, Europe, was used to perform the statistical analysis of the data under study (Informer Technologies, Inc., Hamburg, Germany).

## 3. Results

### 3.1. Sociodemographic and Anthropometric Characteristics of the Study Population

All the sociodemographic and anthropometric parameters of the study population are presented in Table 1. The mean age of the enrolled participants was 37.6 ± 5.8 (range: 21–65) years old. Regarding individuals’ sex, 50.5% were women and 49.5% were men. The mean value of the educational years was 12.2 ± 2.8 (range: 0–14 years). Concerning the financial level, 71.6% of the participants stated a low yearly income, 18.6% a medium- and 9.8% a high yearly income. In total, 85.7% of the participants were Greek and 14.3% of the participants reported another nationality. Concerning living status, 73.1% lived with others and 26.9% lived alone. A total of 80.8% of the participants reported that they were employed and 19.2% of them were unemployed. A total of 70.2% of the enrolled individuals were non-smokers, and the remaining 29.8% were regular smokers. As far as the type of residency, 74.9% lived in urban regions and 25.1% lived in rural regions.

BMI values were normally distributed, and the mean BMI value was 26.9 ± 4.4 (range: 18.2–41.3). According to BMI classification, 65.5% of the assigned participants were classified as normal weight, 21.3% of them as overweight, and 13.2% as obese. Based on the WHR, an indicator of abdominal obesity, 64.2% of the enrolled participants had a low WHR, 23.1% of them had a moderate WHR, and 18.2% exhibited a high WHR.

### 3.2. Lifestyle Factors of the Study Population

All the lifestyle factors are presented in Table 1. Concerning the physical activity levels of the enrolled individuals, according to IPAQ categorization, 58.7% exhibited low physical activity levels, 23.1% showed intermediate physical activity levels, and 18.2% exhibited high physical activity levels. Regarding the sleep quality of the study population, according to PSQI categorization, 69.7% of the enrolled individuals showed adequate sleep quality and 30.3% exhibited inadequate sleep quality. Concerning the quality of life of the assigned participants, according to WHOQOL-Bref, a mean value of 52 ± 2.8 was recorded, while 50.3% had a score below the mean value and 49.7% exhibited a score above the mean value.

Concerning the anxiety of the enrolled participants, based on the six-item short-form STAI classification, 73.4% of the had no symptoms of anxiety, whereas 26.6% of them were diagnosed with anxiety. Regarding the depression of the assigned individuals, according to the BDI-II classification, 66.7% of them had no depressive symptoms, whereas 33.3% of them were diagnosed with depression.

MD compliance was evaluated using MedDietScore. The MedDietScore variable was normally distributed according to the Kolmogorov–Smirnov test, showing a mean value of 27.5 ± 4.8 points (range: 10–43 points). Participants were grouped into quartiles according to MedDietScore [36,37,50,51]. Individuals with a score ≤ 25 (24.9%) were categorized to have “very low” MD compliance and those with scores between 26 and 28 (25.1%) were classified in the “low” MD compliance group. Individuals presenting scores between 29 and 31 (25.0%) were categorized to have “moderate” MD compliance, while those presenting a score ≥ 32 (25.0%) were pointed to have “high” MD compliance [36,37,50,51]. 

### 3.3. Association of MD Adherence with Sociodemographic and Anthropometric Characteristics of the Study Population

In cross-tabulation, elevated MD compliance was significantly more frequently noted in female than male participants (Table 2, *p* = 0.0001). Individuals with a better family annual income exhibited significantly higher levels of MD compliance (Table 2, *p* = 0.0002). Individuals living with others showed considerably elevated levels of MD compliance compared with those living alone (Table 2, *p* = 0.0184). Greater MD compliance was significantly more frequently noted in non-smokers compared with individuals who smoked (Table 2, *p* = 0.0001). Individuals living in rural geographical areas exhibited significantly higher MD compliance than those living in urban areas (Table 2, *p* = 0.0128).

Based on BMI classification, individuals affected by overweight or obesity had significantly lower levels of MD compliance than individuals at target weights (Table 2, *p* = 0.0297). Based on WHR classification, a significantly higher frequency of abdominal obesity was observed in participants adopting the MD at lower levels of compliance than those presenting greater MD compliance (Table 2, *p* = 0.0001). 

A marginal relation of greater MD adherence and advanced education status of participants was noted, though at a non-significant level (Table 2, *p* = 0.0538). Accordingly, employed participants showed a trend of correlation of higher MD adherence compared with unemployed participants, though at a non-significant level (Table 2, *p* = 0.0894). No relation between MD adherence and participants’ age and nationality was noted (Table 2, *p* ˃ 0.05). 

### 3.4. Association of MD Adherence with Lifestyle Factors of the Study Population

Participants with greater MD compliance showed significantly higher physical activity levels (Table 2, *p* = 0.0021). Participants presenting adequate sleep quality showed significantly elevated MD compliance compared with those with inadequate sleep quality (Table 2, *p* = 0.0001). Elevated MD compliance was significantly more frequently observed in individuals with a better quality of life than those with a worse quality of life (Table 2, *p* = 0.0001). A considerably elevated frequency of anxiety symptoms was noted in individuals with lower MD compliance compared with those with higher MD adherence (Table 2, *p* = 0.0001). Accordingly, a considerably elevated incidence of depressive symptoms was observed in individuals with lower MD compliance compared with those with elevated MD adherence (Table 2, *p* = 0.0001).

### 3.5. Multivariate Analysis for MD Adherence with Adjustment for Multiple Confounding Factors

In multiple binary logistic regression analysis, MD compliance was independently associated with participants’ economic level, smoking habits, WHR, physical activity, sleep quality, quality of life, anxiety, and depression. (Table 3, *p* ˂ 0.05). Specifically, female individuals exhibited a 35% higher probability of greater MD compliance compared with male participants (Table 3, *p* = 0.0087). Participants with a high annual income exhibited a 32% greater prevalence of elevated MD compliance compared with those with low or medium annual incomes (Table 3, *p* = 0.0376). Non-smoker individuals showed a 58% higher incidence of greater MD compliance compared with individuals who smoked (Table 3, *p* = 0.0189). Moreover, individuals with lower MD adherence showed a 69% elevated probability to have abdominal obesity (expressed by WHR) compared with those with elevated MD compliance (Table 3, *p* = 0.0184).

In addition, individuals with greater MD compliance showed a 55% greater probability of higher physical activity levels compared with those presenting lower levels of MD adherence (Table 3, *p* = 0.0201). Individuals adopting the MD at higher levels showed a 96% higher prevalence of adequate sleep quality than those presenting reduced MD compliance (Table 3, *p* = 0.0119). Individuals with greater MD compliance exhibited a 2-fold higher incidence of better quality of life than those presenting lower MD adherence levels (Table 3, *p* = 0.0098). Individuals with lower MD compliance had a more than 2-fold elevated probability of being diagnosed with anxiety than those with greater MD adherence (Table 3, *p* = 0.0107). Accordingly, participants with lower MD compliance exhibited a more than 2-fold increased probability of being diagnosed with depression than those with greater MD adherence (Table 3, *p* = 0.0045).

### 3.6. Interrelationships between Sociodemographic, Anthropometric, and Lifestyle Factors

We have further assessed potential interrelationships between the collected data. In fact, overweight and obesity was significantly associated with inadequate sleep quality, urban type of residency, worse quality of life, lower physical activity levels, a higher prevalence of anxiety and depression, and smoking habits (*p* < 0.05). Higher physical activity levels were associated with better sleep quality, urban type of residency, greater quality of life, and lower prevalence of anxiety and depression, as well as never smoking (*p* ˂ 0.05). Better sleep quality was also significantly associated with a lower prevalence of anxiety and depression and not smoking (*p* ˂ 0.05). A better quality of life was also significantly associated with a lower prevalence of anxiety and depression, as well as not smoking (*p* ˂ 0.05). Anxiety was also significantly associated with a higher prevalence of depression and smoking habits (*p* ˂ 0.05).

## 4. Discussion

This is one of the few currently available studies that has explored the impact of MD adherence on multiple sociodemographic, anthropometric, and lifestyles factors during the COVID-19 pandemic. This study has provided evidence that higher levels of MD compliance were independently related to female gender, better economic status, not smoking, a lower risk of abdominal obesity, higher physical activity levels, greater prevalence of adequate sleep quality, better quality of life, and a lower risk of anxiety and depressive symptoms during the COVID-19 pandemic. Associations between greater MD adherence and higher educational levels, living with others, living in rural regions, and a lower prevalence of overweight/obesity were also recorded in the unadjusted analysis. However, these associations were significantly attenuated and did not remain significant after adjustment for several confounders.

According to a recent study published in 2022, 35.4% of Greek adults are overweight and 17.8% are obese. In our study, we recorded a lower prevalence of 21.3% of overweight as well as a bit smaller prevalence of 13.2% of obesity concerning our study population. This may be ascribed to the fact that the mean age of the enrolled individuals in our study was quite low (37.6 ± 5.8 years.), whereas the mean age of the above Greek statistics corresponds to a mean age of about 45 years [54]. The mean value of the educational years was 12.2 ± 2.8 for our study population, which is similar to the official statistics [54]. The prevalence of unemployed individuals in our study population was 19.2%, which is a bit lower compared with the recent official statistics (26.4%) [54]. Food insecurity has been recorded as 72.7% according to the official statistics, which is similar to the percentage of our participants that have reported a 72.9% low and medium financial status. We have also recorded a prevalence of 29.8% of enrolled individuals who were smokers, which is very close to the percentage of 29.0% reported in other studies for Greece [55]. In our study, we recorded a prevalence of 58.7% of the enrolled participants with low physical activity levels, whereas other studies have reported an even higher prevalence of 83% in Greece [56]. Similar decreases in the prevalence of depression, anxiety, stress, poor sleep, and quality of life have also been noted in previous studies in the Greek population [57,58].

There are several studies showing a high prevalence of fatigue, situational and structural anxiety, and depression (60.4%, 60.1%, 46.8%, and 39.7%, respectively) [59,60,61]. Our results also showed that Greek nurses had among the highest rates of fatigue [9,12,41], anxiety, and depression compared with the findings of studies in other countries [6,10,11]. Our results have indicated slightly smaller prevalences of anxiety and depression, 33.3% and 26.6%, which may be ascribed to the fact that we did not focus our study on healthcare professionals, as in previous studies. Greece has the lowest ratio of nurses per 1000 inhabitants among European Union countries [30], a finding that partly justifies these findings [59].

There is substantial research evidence which supports that several phenolic compounds included in the MD exert protective effects against several human diseases, including COVID-19 [27,28,29,30,31]. These phenolic compounds can act against oxidative stress and inflammatory conditions and could enhance human health promotion; they are also considered as preventive and/or co-treatment agents against several human diseases, including COVID-19 infection. Specifically, hydroxytyrosol, resveratrol, flavonols, flavanols, and flavanones may exhibit prevention effects against several human diseases, including COVID-19 [27,28,29,30,31]. All these factors, which are also included in the MD, can support overall human health. The above could be ascribed to the increase in the biosynthesis and translocation of nuclear factor erythroid 2-related factor 2 (Nrf-2), which can enhance the activity of enzymes, which can act against oxidative stress. The above decreases reactive oxygen species (ROS) release and inhibits the action of matrix metalloproteinase-9 (MMP-9), which contributes to the production of cytokines, suppressing the nuclear factor kappa B (NF-κB) [62]. On the contrary, Westernized dietary patterns are commonly associated with increased inflammatory conditions, increased oxidation conditions, and reduced immune system function, and therefore could increase the intensity of COVID-19 symptoms [63,64]. The above detrimental actions may be ascribed to the fact that these dietary patterns contain high amounts of saturated fat, refined carbohydrates, and sugar, whereas they include lower amounts of fibers. Moreover, these dietary patterns have been related to a higher risk of several disorders such as metabolic syndrome and cardiovascular diseases, cancer, and others [63,64]. To date, the currently available epidemiological data remain extremely scarce concerning the impact of healthy dietary patterns like MD against COVID-19. In this context, Greene et al. have demonstrated an inverse association of MD compliance with COVID-19-infected individuals and associated deaths, supporting evidence that the MD and other healthy nutritional patterns, which can decrease inflammatory conditions, also reducing the probability of various pathological states and disorders, may minimize the likelihood of developing intense COVID-19 symptomatology and mortality [65].

A cross-sectional survey including 961 university students has explored their eating habits, and found that the students who were regular smokers and drank high amounts of alcohol have exhibited reduced compliance to the MD. In addition, lower levels of MD compliance were observed in those students with elevated stress and lower sleep quality [66]. Another study has indicated that the quarantine has exerted considerably negative effects on eating behaviors and physical activity [67]. The above has been attributed to the fact that there has been an elevation in food intake and a decrease in physical activity, which can result in body weight elevation [67]. In a longitude study conducted on 1520 participants, increased intake of cereals was related to decreased risk of COVID-19 infection, while the probability of being infected with COVID-19 reduced in combination with increasing olive oil consumption [68]. These findings have supported evidence that adopting a well-balanced MD may be considered as a crucial factor to decrease the likelihood of COVID-19 morbidity [69].

A cross-sectional survey has indicated that healthcare workers have showed a worsening in several aspects of their mental health during the COVID-19 pandemic, leading to increased risk of depressive symptoms, anxiety, and stress [70]. The intensity of the stress and anxiety symptomatology was dependent on the specialization of the healthcare personnel and the duration of exposure with individuals infected with COVID-19. Meanwhile, the intensity of depressive symptoms was dependent on the specific characteristics of each individual infected with COVID-19 [71]. The COVID-19 lockdown has also been associated with a decrease in sleeping hours and physical activity [72]. A cross-sectional survey including 1008 individuals showed that the overall prevalence of depression reached an extremely high percentage of 62.5% [73]. Notably, having depression was significantly associated with anxiety and economic loss, as well as a lower quality of life [73]. In a cross-sectional survey conducted on 1008 Macau residents, the overall prevalence of insomnia was also extremely high, reaching 49.0% during the COVID-19 epidemic period [73]. The above survey has showed that being quarantined in the COVID-19 epidemic period and having psychiatric problems have increased the risk of insomnia and the prevalence of worse quality of life [74]. In addition, a cross-sectional survey including 191 female students has showed an extremely high prevalence of poor sleep quality equal to 82%, also reporting macronutrient imbalances and increased amounts of protein and fat consumption and a decrease in the consumption of carbohydrates and fibers [75]. Another study was performed on 1388 university students. Among them, 66.4% were Lithuanians and 33.6% were Croatians. This study has found a considerably low consumption of vegetables, olive oil, fruits, nuts, legumes, and seafoods during the COVID-19 pandemic, while greater compliance to the MD was related to better physical activity [75].

In an online study conducted on 3797 participants, the MD has decreased the risk of developing anxiety, whereas female gender, worsening of diet quality during the epidemic, and unemployment have enhanced the risk of developing anxiety [76]. Another two retrospective studies from Italy performed on 1401 participants have also supported evidence that psychological distress due to COVID-19 confinement was clearly related to unhealthy nutritional habits in both Italian cohorts [76]. Consumption of ultra-processed foodstuffs was also directly related to a high risk of depression, anxiety, stress, and post-traumatic stress disorder [77]. Notably, a large population-based study performed in Italy during the first COVID-19 lockdown showed that 25.5% and 22% of survey respondents suffered from moderate or severe psychological distress, respectively [75]. Younger age, female gender, being unemployed, and being a student were considerably associated with more intense depression symptomatology [78]. Accordingly, in a cross-sectional, online study performed on 3797 persons, more than half (54%) of the enrolled individuals documented at least mild anxiety, and 25% of them showed intermediate anxiety or even more intense anxiety levels [78]. Remarkably, the MD has a decreased risk of developing intense anxiety levels, even after adjusting for age, gender, and other covariates [78]. However, it should be noted that both healthy dietary and lifestyle habits and not just lifestyle factor, including physical activity levels, daily quality of life, anxiety, stress, depression, smoking habits, and sleep quality, in combination with MD adherence, may exert positive effects on human health.

An observational follow-up study has assessed mental well-being variables in both the pre-lockdown and post-lockdown periods. This study clearly showed that in the post-lockdown period, a worsening in several aspects of life satisfaction occurred [79]. Moreover, a similar worsening effect was recorded concerning depression, quality of life, pain intensity, emotional behavior, and mental health, as well as a deteriorating effect on sleep quality, sleep latency, sleep disturbances, and total sleep quality scoring [79]. In addition, adherence to the MD was found to exert a preventive effect opposed to the enhanced frequency of depressive symptoms [80]. In addition, a prospective study examined the association of depression and anxiety symptomatology determined prior to and in the first weeks of the COVID-19 pandemic with the prevalence of persistent symptomatology in 25,114 individuals and showed that the presence of depression symptoms in the first weeks of the pandemic was related to incident persistent symptoms in both infected and non-infected enrolled individuals [81]. These findings have suggested that depression and anxiety symptomatology must be explored as a potential target for protective interventions against persistent symptomatology due to COVID-19 infection [81]. In another cross-sectional survey, the incidence of depression, anxiety, and stress was shown to be extremely high, reaching 65.7%, 78.5%, and 61.4% of the study population, respectively [82]. Diverse factors have been shown to be related to mental health problems in people with disabilities, such as gender (male), marital state (being married), decreased educational status, comorbid medical diseases, low sleep quality, living in rural regions, hearing disability, disability onset later in life, and testing positive for COVID-19 [82].

Overall, the currently available evidence is in accordance with our results, supporting the fact that nutritional state may be associated with the probability of being infected by COVID-19, and is also related to disease progression. The intake of energy-rich foodstuffs such as sweets, cookies, and cakes has been enhanced in the period of lockdown. An increased prevalence of uncontrolled eating and snacking between meals were also noted during lockdown. 

The COVID-19 pandemic has exerted several negative effects on eating behaviors and the nourishment status of the general population worldwide, increasing the risk of developing diverse mental health disorders like depression, anxiety, and stress. The above could elevate the probability of people’s morbidity from cardiometabolic disorders, inflammation-related pathophysiological states, cancer, sleep disturbances, depression, and anxiety symptomatology. In this aspect, our study has provided evidence that elevated MD compliance could enhance physical activity, sleep quality, and quality of life and may also reduce the risk of developing depression and anxiety symptoms in the period of the COVID-19 pandemic, in accordance with previous studies’ results.

The current survey has several strengths, as it included an adequate representative sample of individuals enrolled from diverse regions of Greece, including both urban and rural areas. The sample size of our survey was quite large and included only Caucasian adults living in 10 geographically diverse Greek regions, and, thus, its representativeness may be recognized as quite adequate. Hence, the present findings may be generalized in other Caucasian European populations of other nationalities. Moreover, our survey is one of the few surveys that investigated the relation of MD compliance with multiple sociodemographic, anthropometric, and lifestyle factors during the COVID-19 pandemic. Another advantage of our survey is that face-to-face interviews between the participating individuals and the qualified personnel were performed to reduce recall bias. The detailed explaining of guidelines and the thorough demonstration of the questions that were systematically provided during the face-to-face interviews may also minimize potential recall bias. Moreover, our study population contained only healthy individuals with no history of any severe disease, with an equal number of women and men. We also examined whether MD adherence during the COVID-19 pandemic may exert independent effects on several sociodemographic, anthropometric, and lifestyle factors by adjusting for numerous potential confounding factors. Finally, we used certified and validated questionnaires which are currently considered as the gold standards for screening physical activity, sleep quality, quality of life, anxiety, and depression, including the IPAQ, PSQI, WHOQOL-Bref, STAI-6, and BDI-II, respectively.

The understanding of the current results should also be taken into consideration with some limitations in mind. The cross-sectional design of this study reduces the likelihood of etiological conclusions and suffers from the possible risk of recall biases, especially for self-reported questions, even if we performed face-to-face interviews. Thus, no definitive conclusions concerning causality should be derived because of our study design. However, self-reported data have been extensively applied in epidemiological studies, showing great consistency and validity to predict several outcomes. Another disadvantage of our survey concerns the fact that BMI and WHR were utilized to distinguish participants’ overweight and obesity as well as abdominal obesity, respectively. Nevertheless, direct determinations of body fatty mass and distribution should be performed to expand and validate the present results. In addition, there is the possibility for unmeasured confounders, even if we have thoroughly adjusted for several confounding factors. Hence, it remains possible that residual confounding factors could affect the present findings. Moreover, our study population is young enough with a mean age of 37.6 ± 5.8 years, which limited our results in young-aged individuals. In this aspect, further studies should be performed in specific different age group populations, e.g., older adults, children, adolescents, in order for more precise conclusion to be drawn. In addition, in our study, a higher prevalence of low income (71.6%) was recorded, which may reflect the young age of our study population. Accordingly, 21.3% of our participants were affected by overweight and 13.2% were affected by obesity, whereas the European TackSHS project showed even more increased prevalence of overweight (33.1%) and obesity (19.7%) [83]. In addition, another limitation of our study is the absence of data concerning serum vitamin D concentrations in our study population. Vitamin D deficiency has been associated with more severe lung involvement, longer disease duration, and risk of death in elderly COVID-19 patients, and vitamin D could be used as supplementary, effective therapy, which may ameliorate and/or treat the symptoms and severity of COVID-19 infection [18,19,20,21,22]. We also used the MD as a healthy dietary pattern to explore the potential presence of any association with the COVID-19 pandemic. However, there are also other healthy dietary patterns that should be investigated in association with the COVID-19 pandemic. A last limitation of our study is that we have not recorded alcohol over-drinking during the COVID-19 pandemic. The COVID-19 pandemic created an environment wherein stress and isolation could increase alcohol consumption. There are several studies that have demonstrated that alcohol consumption was considerably increased during the COVID-19 pandemic, which may increase the risk of depression, anxiety, stress, and other lifestyle factors in daily routines [84]. Substantial studies have also provided evidence that chronic alcohol consumption and alcohol use disorder have increased the risk of COVID-19 infection and severe disease progression [85].

## 5. Conclusions

The COVID-19 pandemic has had important detrimental consequences on several aspects of mental health, beyond the detrimental effects on public health and quality of life. MD adherence seems to improve several aspects of COVID-19 complications, like depression, anxiety, sleep quality, physical activity, and quality of life, within daily life. However, the currently available studies on this topic remain extremely scarce. Thus, it is strongly recommended to perform further well-designed and population-based studies to assess the potential benefits of diverse healthy dietary patterns, including the MD, against COVID-19 complications, with the aim to establish reliable and conclusive results.

## Figures and Tables

**Table 1:** Descriptive statistics of the study population.

Parameters (n = 3721)	Descriptive Statistics
**Age (mean ± SD; years)**	37.6 ± 5.8
**Gender (n, %)**	
Male	1841 (49.5%)
Female	1880 (50.5%)
**Education level (mean ± SD; years)**	12.2 ± 2.8
**Family financial status (n, %)**	
Low	2664 (71.6%)
Medium	693 (18.6%)
High	364 (9.8%)
**Nationality (n, %)**	
Greek	3189 (85.7%)
Other	531 (14.3%)
**Living status (n, %)**	
Living with others	2720 (73.1%)
Living alone	1001 (26.9%)
**Employment (n, %)**	
Employed	3006 (80.8%)
Unemployed	715 (19.2%)
**Smoking habits (n, %)**	
Non-smokers	2612 (70.2%)
Smokers	1109 (29.8%)
**Type of residency** **(n, %)**	
Urban	2789 (74.9%)
Rural	932 (25.1%)
**BMI status (n, %)**	
Normal weight	2437 (65.5%)
Overweight	792 (21.3%)
Obese	492 (13.2%)
**WHR (n, %)**	
Low	2387 (64.2%)
Moderate	854 (22.9%)
High	480 (12.9%)
**Physical activity levels (n, %)**	
Low	2186 (58.7%)
Moderate	859 (23.1%)
High	676 (18.2%)
**Sleep quality (n, %)**	
Adequate	2595 (69.7%)
Inadequate	1126 (30.3%)
**Quality of life (n, %)**	
Below mean value	1872 (50.3%)
Over mean value	1849 (49.7%)
**Anxiety (n, %)**	
No	2731 (73.4%)
Yes	990 (26.6%)
**Depression (n, %)**	
No	2482 (66.7%)
Yes	1239 (33.3%)
**Mediterranean Diet adherence (n, %)**	
Very low	927 (24.9%)
Low	932 (25.1%)
Moderate	931 (25.0%)
High	931 (25.0%)

**Table 2:** Association of MD adherence with sociodemographic, anthropometric, and lifestyle factors.

Characteristics (n = 3721)	Mediterranean Diet Adherence				
	Very Low	Low	Moderate	High	*p*-Value
**Age (mean ± SD; years)**	37.5 ± 9.1	37.2 ± 8.8	37.9 ± 8.5	37.6 ± 8.0	*p* = 0.3857
**Gender (n, %)**					
Male	499 (53.8%)	638 (68.5%)	355 (38.1%)	348 (37.4%)	*p* = 0.0001
Female	428 (46.2%)	294 (31.5%)	576 (61.9%)	583 (62.6%)	
**Education level (mean ± SD; years)**	12.8 ± 4.5	12.7 ± 4.3	13.1 ± 4.8	13.3 ± 5.1	*p* = 0.0538
**Family financial status (n, %)**					*p* = 0.0002
Low	704 (75.9%)	689 (73.9%)	724 (77.8%)	547 (58.7%)	
Medium	187 (29.2%)	206 (22.1%)	168 (18.0%)	132 (14.2%)	
High	36 (3.9%)	37 (4.0%)	39 (4.2%)	252 (27.1%)	
**Nationality (n, %)**					*p* = 0.4538
Greek	794 (85.6)	789 (84.7%)	811 (87.2%)	795 (85.4%)	
Other	133 (14.4%)	143 (15.3%)	119 (12.8%)	136 (14.6%)	
**Living status (n, %)**					*p* = 0.0184
Living with others	657 (70.9%)	711 (76.3%)	643 (69.1%)	709 (76.2%)	
Living alone	270 (29.1)	221 (23.7%)	288 (30.9%)	222 (23.8%)	
**Employment (n, %)**					*p* = 0.0894
Employed	730 (78.7%)	764 (82.0%)	747 (80.2%)	765 (82.2%)	
Unemployed	197 (21.3%)	168 (18.0%)	184 (19.8%)	166 (17.8%)	
**Smoking habits (n, %)**					*p* = 0.0001
Non-smokers	593 (64.0%)	528 (56.7%)	723 (77.7%)	768 (82.5%)	
Smokers	334 (36.0%)	404 (43.3%)	208 (22.3%)	163 (17.5%)	
**Type of residency (n, %)**					*p* = 0.0128
Urban	744 (80.3%)	692 (74.3%)	689 (74.0%)	664 (71.3%)	
Rural	183 (19.7%)	240 (25.7%)	242 (26.0%)	267 (28.7%)	
**BMI status (n, %)**					*p* = 0.0297
Normal weight	607 (65.5%)	575 (61.7%)	621 (66.7%)	634 (68.1%)	
Overweight	182 (19.6%)	225 (24.1%)	201 (21.6%)	184 (19.8%)	
Obese	138 (14.9%)	132 (14.2%)	109 (11.7%)	113 (12.1%)	
**WHR (n, %)**					*p* = 0.0001
Low	576 (62.1%)	544 (58.4%)	586 (62.9%)	681 (73.2%)	
Moderate	223 (24.1%)	260 (27.9%)	219 (23.5%)	152 (16.3%)	
High	128 (13.8%)	128 (13.7%)	126 (13.5%)	98 (10.5%)	
**Physical activity levels (n, %)**					*p* = 0.0021
Low	750 (80.9%)	766 (82.2%)	334 (35.9%)	336(36.1%)	
Moderate	163 (17.6%)	157 (16.8%)	344 (36.9%)	195 (20.9%)	
High	14 (1.51%)	9 (1.0%)	253 (27.2%)	400 (43.0%)	
**Sleep quality (n, %)**					*p* = 0.0001
Adequate	539 (58.1%)	525 (56.3%)	758 (81.4%)	773 (83.0%)	
Inadequate	388 (41.9%)	407 (43.7%)	173 (18.6%)	158 (17.0%)	
**Quality of life (n, %)**					*p* = 0.0001
Below mean value	458 (49.4%)	598 (64.2%)	491 (52.7%)	325 (34.9%)	
Over mean value	469 (50.6%)	334 (35.8%)	440 (47.3%)	606 (65.1%)	
**Anxiety (n, %)**					*p* = 0.0001
No	610 (65.8%)	642 (68.9%)	700 (75.2%)	779 (83.7%)	
Yes	317 (34.2%)	290 (31.1%)	231 (24.8%)	152 (16.3%)	
**Depression (n, %)**					*p* = 0.0001
No	530 (57.2%)	573 (61.5%)	661 (71.0%)	718 (77.1%)	
Yes	397 (42.8%)	359 (38.5%)	270 (29.0%)	213 (22.9%)	

**Table 3:** Multivariate analysis for MD adherence, adjusting for possible confounders.

Characteristics	Mediterranean Diet Adherence (Very Low + Low vs. Moderate + High)	
	OR * (95% CI **)	*p*-Value
**Gender** (Male/Female)	1.35 (1.03–1.57)	*p* = 0.0087
**Educational status** (Below/Over mean value)	1.28 (0.77–1.82)	*p* = 0.2938
**Family financial level** (Low or medium/High)	1.32 (0.98–1.65)	*p* = 0.0376
**Nationality** (Greek /Other)	0.91 (0.23–1.67)	*p* = 0.8932
**Living status** (Living alone/Living with others)	1.42 (1.05–1.87)	*p* = 0.0783
**Employment** (Unemployed/Employed)	1.15 (0.71–1.68)	*p* = 0.2394
**Smoking habits** (Yes/No)	1.58 (1.33–1.84)	*p* = 0.0189
**Type of residency** (Urban/Rural)	1.10 (0.69–1.58)	*p* = 0.1947
**BMI status** (Normal weight/Overweight + Obese)	1.87 (1.28–2.43)	*p* = 0.2043
**WHR** (Moderate + high/Low)	1.69 (1.42–1.81)	*p* = 0.0184
**Physical activity levels** (Low/Moderate + high)	1.55 (1.18–1.89)	*p* = 0.0201
**Sleep quality** (Inadequate/Adequate)	1.96 (1.72–2.29)	*p* = 0.0119
**Quality of life** (Below mean value/Over mean value)	2.04 (1.83–2.27)	*p* = 0.0098
**Anxiety** (Yes/No)	2.18 (1.93–2.41)	*p* = 0.0107
**Depression** (Yes/No)	2.43 (2.25–2.66)	*p* = 0.0045

* Odds Ratio: OR. ** CI: Confidence interval.

## Data Availability

Data are available upon request from the corresponding author.

## References

[1] Almubarak S.H., Alsaif A.K., Almulla S.J., Alfayez A.S., Alnujaidi H.Y., Alsalman D.M. (2023). Teleworking during COVID-19: Ex-periences from Saudi Arabia. Ind. Health.

[2] Pérez-Cano H.J., Moreno-Murguía M.B., Morales-López O., Crow-Buchanan O., English J.A., Lozano-Alcázar J., Somilleda-Ventura S.A. (2020). Anxiety, depression, and stress in response to the coronavirus disease-19 pandemic. Cir. Cir..

[3] Fernández-Aranda F., Casas M., Claes L., Bryan D.C., Favaro A., Granero R., Gudiol C., Jiménez-Murcia S., Karwautz A., Le Grange D. (2020). COVID-19 and implications for eating disorders. Eur. Eat. Disord. Rev..

[4] De Hert M., Mazereel V., Detraux J., Van Assche K. (2021). Prioritizing COVID-19 vaccination for people with severe mental illness. World Psychiatry.

[5] Sideli L., Lo Coco G., Bonfanti R.C., Borsarini B., Fortunato L., Sechi C., Micali N. (2021). Effects of COVID-19 lockdown on eating disorders and obesity: A systematic review and meta-analysis. Eur. Eat. Disord. Rev..

[6] Wang Y., Di Y., Ye J., Wei W. (2021). Study on the public psychological states and its related factors during the outbreak of coronavirus disease 2019 (COVID-19) in some regions of China. Psychol. Health Med..

[7] Machado P.P.P., Pinto-Bastos A., Ramos R., Rodrigues T.F., Louro E., Gonçalves S., Brandão I., Va A. (2020). Impact of COVID-19 lockdown measures on a cohort of eating disorders patients. J. Eat. Disord..

[8] Galea S., Merchant R.M., Lurie N. (2020). The Mental Health Consequences of COVID-19 and Physical Distancing: The Need for Prevention and Early Intervention. JAMA Intern. Med..

[9] Ferrante G., Camussi E., Piccinelli C., Senore C., Armaroli P., Ortale A., Garena F., Giordano L. (2020). Did social isolation during the SARS-CoV-2 epidemic have an impact on the lifestyles of citizens?. Epidemiol. Prev..

[10] Mrityunjaya M., Pavithra V., Neelam R., Janhavi P., Halami P.M., Ravindra P.V. (2020). Immune-Boosting, Antioxidant and Anti-Inflammatory Food Supplements Targeting Pathogenesis of COVID-19. Front. Immunol..

[11] Lordan R., Rando H.M., Greene C.S. (2021). Dietary Supplements and Nutraceuticals under Investigation for Covid-19 Prevention and Treatment. Msystems.

[12] Mortaz E., Bezemer G., Alipoor S.D., Varahram M., Mumby S., Folkerts G., Garssen J., Adcock I.M. (2021). Nutritional Impact and Its Potential Consequences on Covid-19 Severity. Front. Nutr..

[13] Paces J., Strizova Z., Smrz D., Cerny J. (2020). COVID-19 and the Immune System. Physiol. Res..

[14] Tyrovolas S., Giné-Vázquez I., Fernández D., Morena M., Koyanagi A., Janko M., Haro J.M., Lin Y., Lee P., Pan W. (2021). Estimating the COVID-19 Spread through Real-Time Population Mobility Patterns: Surveillance in Low- and Middle-Income Countries. J. Med. Internet Res..

[15] Beigmohammadi M.T., Bitarafan S., Hoseindokht A., Abdollahi A., Amoozadeh L., Soltani D. (2021). The effect of supplementation with vitamins A, B, C, D, and E on disease severity and inflammatory responses in patients with COVID-19: A randomized clinical trial. Trials.

[16] Karonova T.L., Chernikova A.T., Golovatyuk K.A., Bykova E.S., Grant W.B., Kalinina O.V., Grineva E.N., Shlyakhto E.V. (2022). Vitamin D Intake May Reduce SARS-CoV-2 Infection Morbidity in Health Care Workers. Nutrients.

[17] Shakoor H., Feehan J., Al Dhaheri A.S., Ali H.I., Platat C., Ismail L.C., Apostolopoulos V., Stojanovska L. (2021). Immune-Boosting Role of Vitamins D, C, E, Zinc, Selenium and Omega-3 Fatty Acids: Could They Help against Covid-19?. Maturitas.

[18] Majidi N., Rabbani F., Gholami S., Gholamalizadeh M., BourBour F., Rastgoo S., Hajipour A., Shadnoosh M., Akbari M.E., Bahar B. (2021). The Effect of Vitamin C on Pathological Pa-rameters and Survival Duration of Critically Ill Coronavirus Disease 2019 Patients: A Randomized Clinical Trial. Front. Immunol..

[19] Ghelani D., Alesi S., Mousa A. (2021). Vitamin D and COVID-19: An Overview of Recent Evidence. Int. J. Mol. Sci..

[20] Rastogi A., Bhansali A., Khare N., Suri V., Yaddanapudi N., Sachdeva N., Puri G.D., Malhotra P. (2022). Short term, high-dose vita-min D supplementation for COVID-19 disease: A randomised, placebo-controlled, study (SHADE study). Postgrad Med. J..

[21] Cutolo M., Paolino S., Sulli A., Smith V., Pizzorni C., Seriolo B. (2014). Vitamin D, steroid hormones, and autoimmunity. Ann. N. Y. Acad. Sci..

[22] Sulli A., Gotelli E., Casabella A., Paolino S., Pizzorni C., Alessandri E., Grosso M., Ferone D., Smith V., Cutolo M. (2021). Vitamin D and Lung Outcomes in Elderly COVID-19 Patients. Nutrients.

[23] Cutolo M., Smith V., Paolino S., Gotelli E. (2023). Involvement of the secosteroid vitamin D in autoimmune rheumatic diseases and COVID-19. Nat. Rev. Rheumatol..

[24] Sedighiyan M., Abdollahi H., Karimi E., Badeli M., Erfanian R., Raeesi S., Hashemi R., Vahabi Z., Asanjarani B., Mansouri F. (2021). Omega-3 polyunsaturated fatty acid supplementation improve clinical symptoms in patients with 64 Covid-19: A randomized clinical trial. Int. J. Clin. Pract..

[25] Gutiérrez-Castrellón P., Gandara-Martí T., Abreu A.T.Y., Nieto-Rufino C.D., López-Orduña E., Jiménez-Escobar I., Jimé-nez-Gutiérrez C., López-Velazquez G., Espadaler-Mazo J. (2022). Probiotic improves symptomatic and viral clearance in Covid19 outpa-tients: A randomized, quadruple-blinded, placebo-controlled trial. Gut Microbes.

[26] Brahma S., Naik A., Lordan R. (2022). Probiotics: A gut response to the COVID-19 pandemic but what does the evidence show?. Clin. Nutr. ESPEN.

[27] Yang J., Li X., He T., Ju F., Qiu Y., Tian Z. (2022). Impact of Physical Activity on COVID-19. Int. J. Environ. Res. Public Health.

[28] Kiani A.K., Medori M.C., Bonetti G., Aquilanti B., Velluti V., Matera G., Iaconelli A., Stuppia L., Connelly S.T., Herbst K.L. (2022). Modern vision of the Mediterranean diet. J. Prev. Med. Hyg..

[29] Schwingshackl L., Morze J., Hoffmann G. (2020). Mediterranean diet and health status: Active ingredients and pharmacological mechanisms. Br. J. Pharmacol..

[30] Andreo-López M.C., Contreras-Bolívar V., Muñoz-Torres M., García-Fontana B., García-Fontana C. (2023). Influence of the Mediterranean Diet on Healthy Aging. Int. J. Mol. Sci..

[31] Vetrani C., Piscitelli P., Muscogiuri G., Barrea L., Laudisio D., Graziadio C., Marino F., Colao A. (2022). “Planeterranea”: An attempt to broaden the beneficial effects of the Mediterranean diet worldwide. Front. Nutr..

[32] Finicelli M., Di Salle A., Galderisi U., Peluso G. (2022). The Mediterranean Diet: An Update of the Clinical Trials. Nutrients.

[33] Farrugia F., Refalo D., Bonello D., Cuschieri S. (2023). The impact of the COVID-19 pandemic on Mediterranean diet adherence: A narrative systematic review. Nutr. Health.

[34] Ferro Y., Pujia R., Maurotti S., Boragina G., Mirarchi A., Gnagnarella P., Mazza E. (2021). Mediterranean Diet a Potential Strategy against SARS-CoV-2 Infection: A Narrative Review. Medicina.

[35] Ponzo V., Pellegrini M., D’eusebio C., Bioletto F., Goitre I., Buscemi S., Bo S. (2021). Mediterranean diet and sars-cov-2 infection: Is there any association? A proof-of-concept study. Nutrients.

[36] Perez-Araluce R., Martinez-Gonzalez M.A., Fernández-Lázaro C.I., Bes-Rastrollo M., Gea A., Carlos S. (2022). Mediterranean diet and the risk of COVID-19 in the ‘Seguimiento Universidad de Navarra’ cohort. Clin. Nutr..

[37] Izzo L., Santonastaso A., Cotticelli G., Federico A., Pacifico S., Castaldo L., Colao A., Ritieni A. (2021). An Italian Survey on Dietary Habits and Changes during the COVID-19 Lockdown. Nutrients.

[38] Mantzorou M., Mentzelou M., Vasios G.K., Kontogiorgis C., Antasouras G., Vadikolias K., Psara E., Vorvolakos T., Poulios E., Serdari A. (2023). Mediterranean Diet Adherence Is Associated with Favorable Health-Related Quality of Life, Physical Activity, and Sleep Quality in a Community-Dwelling Greek Older Population. Antioxidants.

[39] Mantzorou M., Vadikolias K., Pavlidou E., Tryfonos C., Vasios G., Serdari A., Giaginis C. (2021). Mediterranean diet adherence is associated with better cognitive status and less depressive symptoms in a Greek elderly population. Aging Clin. Exp. Res..

[40] World Health Organization (2006). The World Health Report: 2006: Working Together for Health.

[41] Jamesm W.P. (2008). WHO recognition of the global obesity epidemic. Int. J. Obes..

[42] World Health Organization (2011). Waist Circumference and Waist-Hip Ratio: Report of a WHO Expert Consultation, Geneva, 8–11 December.

[43] Ahmad N., Adam S.I.M., Nawi A.M., Hassan M.R., Ghazi H.F. (2016). Abdominal Obesity Indicators: Waist Circumference or Waist-to-hip Ratio in Malaysian Adults Population. Int. J. Prev. Med..

[44] Bener A., Yousafzai M.T., Darwish S., Al-Hamaq A.O., Nasralla E.A., Abdul-Ghani M. (2013). Obesity index that better predict met-abolic syndrome: Body mass index, waist circumference, waist hip ratio, or waist height ratio. J Obes..

[45] Yanga F., Lv J.H., Lei S.F., Chena X.D. (2006). Receiver-operating characteristic analyses of body mass index, waist circumference and waist-to-hip ratio for obesity: Screening in young adults in central south of China. Clin. Nut..

[46] Cheng C.H., Ho C.C., Yang C.F., Huang Y.C., Lai C.H., Liaw Y.P. (2010). Waist-to-hip ratio is a better anthropometric index than body mass index for predicting the risk of type 2 diabetes in Taiwanese population. Nutr. Res..

[47] Craig C.L., Marshall A.L., Sjostrom M., Bauman A.E., Booth M.L., Ainsworth B.E., Pratt M., Ekelund U., Yngve A., Sallis J.F. (2003). International physical activity questionnaire: 12-country reliability and validity. Med. Sci. Sport. Exerc..

[48] Salahuddin M., Maru T.T., Kumalo A., Pandi-Perumal S.R., Bahammam A.S., Manzar M.D. (2017). Validation of the Pittsburgh sleep quality index in community dwelling Ethiopian adults. Health Qual. Life Outcomes.

[49] Kalfoss M.H., Reidunsdatter R.J., Klöckner C.A., Nilsen M. (2021). Validation of the WHOQOL-Bref: Psychometric properties and normative data for the Norwegian general population. Health Qual. Life Outcomes.

[50] Tluczek A., Henriques J.B., Brown R.L. (2009). Support for the reliability and validity of a six-item state anxiety scale derived from the State-Trait Anxiety Inventory. J. Nurs. Meas..

[51] Wang Y.-P. (2013). Clarice Gorenstein. Psychometric properties of the Beck Depression Inventory-II: A comprehensive review. Rev. Bras. Psiquiatr..

[52] Panagiotakos D.B., Pitsavos C., Stefanadis C. (2006). Dietary patterns: A Mediterranean diet score and its relation to clinical and bio-logical markers of cardiovascular disease risk. Nutr. Metab. Cardiovasc. Dis..

[53] Arvaniti F., Panagiotakos D.B. (2008). Healthy indexes in public health practice and research: A review. Crit. Rev. Food Sci. Nutr..

[54] Diamantis D.V., Karatzi K., Kantaras P., Liatis S., Iotova V., Bazdraska T., Tankova T., Greet Cardon G., Wikström K., Rurik I. (2022). Prevalence and Socioeconomic Correlates of Adult Obesity in Europe: The Feel4Diabetes Study. Int. J. Environ. Res. Public Health.

[55] Melpo Kapetanstrataki M., Anna Tzortzi A., Vaso Evangelopoulou V., Behrakis P. (2021). Profiling smokers in Greece in 2020. Tob. Pre. Cessat..

[56] Psarrou A., Adamakidou T., Apostolara P., Koreli A., Drakopoulou M., Plakas S., Mastrogiannis D., Mantoudi A., Parissopoulos S., Zartaloudi A. (2023). Associations between Physical Activity and Health-Related Quality of Life among Community-Dwelling Older Adults: A Cross-Sectional Study in Urban Greece. Geriatrics.

[57] Papadopoulou A., Efstathiou V., Yotsidi V., Pomini V., Michopoulos I., Markopoulou E., Papadopoulou M., Tsigkaropoulou E., Kalemi G., Tournikioti K. (2021). Suicidal ideation during COVID-19 lockdown in Greece: Prevalence in the community, risk and protective factors. Psychiatry Res..

[58] Gournellis R., Efstathiou V. (2021). The impact of the COVID-19 Pandemic on the Greek population: Suicidal ideation during the first and second lockdown. Psychiatriki.

[59] Health at a Glance: Europe 2020 State of Health in the EU Cycle. https://ec.europa.eu/health/system/files/2020-12/2020_healthatglance_rep_en_0.pd.

[60] Sikaras C., Zyga S., Tsironi M., Tselebis A., Pachi A., Ilias I., Panagiotou A. (2023). The Mediating Role of Depression and of State Anxiety οn the Relationship between Trait Anxiety and Fatigue in Nurses during the Pandemic Crisis. Healthcare.

[61] Labrague L.J. (2021). Pandemic fatigue and clinical nurses’ mental health, sleep quality and job contentment during the COVID-19 pandemic: The mediating role of resilience. J. Nurs. Manag..

[62] Sasangohar F., Jones S.L., Masud F.N., Vahidy F.S., Kash B.A. (2020). Provider Burnout and Fatigue During the COVID-19 Pandemic: Lessons Learned from a High-Volume Intensive Care Unit. Obstet. Anesthesia Dig..

[63] Milton-Laskibar I., Trepiana J., Macarulla M.T., Gómez-Zorita S., Arellano-García L., Fernández-Quintela A., Portillo M.P. (2023). Potential usefulness of Mediterranean diet polyphenols against COVID-19-induced inflammation: A review of the current knowledge. J. Physiol. Biochem..

[64] Drake I., Sonestedt E., Ericson U., Wallström P., Orho-Melander M. (2018). A western dietary pattern is prospectively associated with cardio-metabolic traits and incidence of the metabolic syndrome. Br. J. Nutr..

[65] Port J.R., Adney D.R., Schwarz B., Schulz J.E., Sturdevant D.E., Smith B.J., Avanzato V.A., Holbrook M.G., Purushotham J.N., Stromberg K.A. (2021). High-Fat high-sugar diet-induced changes in the lipid metabolism are associated with mildly increased COVID-19 severity and delayed recovery in the Syrian hamster. Viruses.

[66] Greene M.W., Roberts A.P., Frugé A.D. (2021). Negative association between Mediterranean diet adherence and COVID-19 cases and related deaths in Spain and 23 OECD countries: An ecological study. Front. Nutr..

[67] Maté-Muñoz J.L., Hernández-Lougedo J., Ruiz-Tovar J., Olivares-Llorente R., García-Fernández P., Zapata I. (2023). Physical Activity Levels, Eating Habits, and Well-Being Measures in Students of Healthcare Degrees in the Second Year of the COVID-19 Pandemic. Healthcare.

[68] Catucci A., Scognamiglio U., Rossi L. (2021). Lifestyle Changes Related to Eating Habits, Physical Activity, and Weight Status During COVID-19 Quarantine in Italy and Some European Countries. Front. Nutr..

[69] Sharma S., Di Castelnuovo A., Costanzo S., Persichillo M., Panzera T., Ruggiero E., De Curtis A., Storto M., Cavallo P., Gianfagna F. (2023). Habitual adherence to a traditional Mediterranean diet and risk of SARS-CoV-2 infection and Coronavirus disease 2019 (COVID-19): A longitudinal analysis. Int. J. Food Sci. Nutr..

[70] Alhouri A., Shokor M.A., Marwa K., Sharabi A., Arrouk D.M.N., Al Houri F.N., Al Houri H. (2023). COVID-19 and Its Impact on Healthcare Workers: Understanding Stigma, Stress, and Quality of Life. Cureus.

[71] Turki S., Bouzekri K., Trabelsi T., El Ati J. (2022). Assessment of Mediterranean Diet Adherence and Lifestyle Change during COVID-19 National Lockdown in Tunisian Adult Population. Nutrients.

[72] Si T.L., Chen P., Zhang L., Sha S., Lam M.I., Lok K.I., Chow I.H.I., Li J.X., Wang Y.Y., Su Z. (2023). Depression and quality of life among Macau residents in the 2022 COVID-19 pandemic wave from the perspective of network analysis. Front. Psychol..

[73] Chen P., Zhang L., Sha S., Lam M.I., Lok K.I., Chow I.H.I., Si T.L., Su Z., Cheung T., Feng Y. (2023). Prevalence of insomnia and its association with quality of life among Macau residents shortly after the summer 2022 COVID-19 outbreak: A network analysis perspective. Front. Psychiatry.

[74] Díaz G., Hernández S., Crespo A., Renghea A., Yébenes H., Iglesias-López M.T. (2023). Macronutrient Intake, Sleep Quality, Anxiety, Adherence to a Mediterranean Diet and Emotional Eating among Female Health Science Undergraduate Students. Nutrients.

[75] Mieziene B., Burkaite G., Emeljanovas A., Tilindiene I., Novak D., Kawachi I. (2022). Adherence to Mediterranean diet among Lithuanian and Croatian students during COVID-19 pandemic and its health behavior correlates. Front. Public Health.

[76] Boaz M., Navarro D.A., Raz O., Kaufman-Shriqui V. (2021). Dietary Changes and Anxiety during the Coronavirus Pandemic: Differences between the Sexes. Nutrients.

[77] Bonaccio M., Costanzo S., Bracone F., Gialluisi A., Di Castelnuovo A., Ruggiero E., Esposito S., Olivieri M., Persichillo M., Cerletti C. (2022). Psychological distress resulting from the COVID-19 confinement is associated with unhealthy dietary changes in two Italian population-based cohorts. Eur. J. Nutr..

[78] Lorenzoni G., Azzolina D., Maresio E., Gallipoli S., Ghidina M., Baldas S., Berchialla P., Giron M.C., Silano M., Gregori D. (2022). Impact of the COVID-19 lockdown on psychological health and nutritional habits in Italy: Results from the #PRESTOinsieme study. BMJ Open.

[79] Kaufman-Shriqui V., Navarro D.A., Raz O., Boaz M. (2022). Dietary changes and anxiety during the coronavirus pandemic: A multinational survey. Eur. J. Clin. Nutr..

[80] Marcos-Pardo P.J., Abelleira-Lamela T., Vaquero-Cristobal R., González-Gálvez N. (2022). Changes in life satisfaction, depression, general health and sleep quality of Spanish older women during COVID-19 lockdown and their relationship with lifestyle: An observational follow-up study. BMJ Open.

[81] Matta J., Robineau O., Wiernik E., Carrat F., Severi G., Touvier M., Gouraud C., Ouazana Vedrines C., Pitron V., Ranque B. (2023). Depression and anxiety before and at the beginning of the COVID-19 pandemic and incident persistent symptoms: A prospective population-based cohort study. Mol. Psychiatry.

[82] Roy N., Amin M.B., Mamun M.A., Sarker B., Hossain E., Aktarujjaman M. (2023). Prevalence and factors associated with depression, anxiety, and stress among people with disabilities during COVID-19 pandemic in Bangladesh: A cross-sectional study. PLoS ONE.

[83] Stival C., Lugo A., Odone A., van den Brandt P.A., Fernandez E., Tigova O., Soriano J.B., López M.J., Scaglioni S., Gallus S. (2022). Prevalence and Correlates of Overweight and Obesity in 12 European Countries in 2017–2018. Obes. Facts.

[84] Calina D., Hartung T., Mardare I., Mitroi M., Poulas K., Tsatsakis A., Rogoveanu I., Docea A.O. (2021). COVID-19 pandemic and alcohol consumption: Impacts and interconnections. Toxicol. Rep..

[85] Friske M.M., Spanagel R. (2023). Chronic alcohol consumption and COVID-19 infection risk: A narrative review. Alcohol. Clin. Exp. Res..

